# Divergent Mononuclear Cell Participation and Cytokine Release Profiles Define Hip and Knee Osteoarthritis

**DOI:** 10.3390/jcm8101631

**Published:** 2019-10-05

**Authors:** Ricardo Grieshaber-Bouyer, Till Kämmerer, Nils Rosshirt, Timo A. Nees, Philipp Koniezke, Elena Tripel, Marcus Schiltenwolf, Johannes Kirsch, Sébastien Hagmann, Babak Moradi

**Affiliations:** 1 Clinic for Orthopaedic and Trauma Surgery, University Clinic of Heidelberg, Schlierbacher, Landstr 200a, 69118 Heidelberg, Germany; Ricardo.GrieshaberBouyer@med.uni-heidelberg.de (R.G.-B.); Till.Kaemmerer@med.uni-heidelberg.de (T.K.); Nils.Rosshirt@med.uni-heidelberg.de (N.R.); Timo.Nees@med.uni-heidelberg.de (T.A.N.); Philipp.Koniezke@med.uni-heidelberg.de (P.K.); Elena.Tripel@med.uni-heidelberg.de (E.T.); Marcus.Schiltenwolf@med.uni-heidelberg.de (M.S.); Johannes.Kirsch@med.uni-heidelberg.de (J.K.); Sebastien.Hagmann@med.uni-heidelberg.de (S.H.); 2 Division of Rheumatology, Immunology, and Allergy, Brigham and Women’s Hospital and Harvard Medical School, Boston, MA 02115, USA

**Keywords:** osteoarthritis, knee, hip, inflammation, synovial membrane, cytokine

## Abstract

Osteoarthritis (OA) is a progressive joint disease driven by a blend of inflammatory and biomechanical processes. Studies using human samples to understand inflammatory mechanisms in OA frequently recruit OA patients with different affected joints, even though recent evidence indicates that OA is a heterogeneous disease which only culminates in a common end point. Differences in age of onset and the dynamics of disease progression suggest that different joints may represent different disease entities, thereby diluting the discovery potential in a combined analysis. We hypothesized that different OA joints may also differ in immunopathology within the synovium. To investigate this hypothesis, we profiled the immune cell contribution (flow cytometry) and cytokine release profiles (ELISA) in purified synovial membrane mononuclear cells from 50 patients undergoing either hip (*n* = 34) or knee (*n* = 16) replacement surgery. Unsupervised computational approaches were used for disease deconstruction. We found that hip and knee osteoarthritis are not identical in respect to the inflammatory processes that take place in the synovial membrane. Instead, we report that principally CD14^+^ macrophages are expanded fourfold in the synovial membrane of patients with knee OA compared to hip OA, with a trend to higher expression in CD8^+^ T cells, while CD4^+^ T cells, B cells, and NK cells were found at comparable quantities. Upon isolation and culture of cells from synovial membrane, isolates from hip OA released higher concentrations of Eotaxin (CCL11), G-CSF, GM-CSF, INF-γ, IP-10 (CXCL10), TNF-α, MIP-1α (CCL3), MIP-1β (CCL4), IL-4, IL-10, IL-17, and lower concentrations of stem cell factor (SCF), thereby highlighting the difference in the nature of hip and knee osteoarthritis. Taken together, this study establishes hip and knee OA as immunologically distinct types of OA, and creates a resource of the cytokine expression landscape and mononuclear cell infiltration pattern of patients with hip and knee osteoarthritis.

## 1. Background

Osteoarthritis (OA) is a heterogeneous disease, and with a prevalence of symptomatic OA of 10% in men and 18% in women aged over 60, represents the most frequent cause of disability in older adults according to the World Health Organization [1]. As a consequence of a growing elderly population, OA prevalence will continuously increase and represent a growing burden to the healthcare infrastructure and economy [2]. As a result, treatment of OA is not only important for the patients’ pain relief and improvement of functional performance, it is also necessary for the economical function of a growing older society. As current therapeutic options act solely symptomatic, identifying promising targets for medical intervention of OA is a major objective of current biomedical research.

OA was traditionally viewed as a non-inflammatory joint disease with ageing, with mechanical stress being considered the major contributors to cartilage breakdown and bone remodeling. This still being relevant, evidence for an inflammatory component to OA pathogenesis is significantly increasing [3,4,5]. The pathogenesis of OA involves a wide array of immune cells and inflammatory mediators. Histopathological hallmarks of osteoarthritis are subchondral bone resorption, cartilage destruction, and synovial hypertrophy [2]. Macrophages and CD4^+^ T helper cells are the two principal cell types in osteoarthritic synovial membrane, while additional effector cells contributing to joint damage are synovial fibroblasts and osteoclasts [3]. Infiltrating immune cells use soluble mediators to communicate with resident cells, the two predominant inflammatory cytokines being IL-1β and TNF-α [2]. In the inflamed synovium, IL-1β and TNF-α act synergistically to alter the metabolic properties of resident chondrocytes and drive the release of enzymes that degrade cartilage matrix (MMP-1, MMP-3, MMP-13) [4,5]. In addition to promoting matrix disintegration, they also act to suppress the production of new matrix by interfering with proteoglycan and type II collagen formation in chondrocytes [6]. IL-1β also restricts aggrecan formation in chondrocytes [7]. IL-17 is another central cytokine in joint inflammation. It is secreted by Th17 cells in response to IL-23 and sustains inflammation by supporting production of further inflammatory cytokines and attracting influx of more immune cells, for example neutrophils. Therefore, inflammatory mediators are implied in OA pathophysiology, and immunological mechanisms in OA are fundamental to study, but still far from understood.

In contrast to traditional belief, osteoarthritis is now believed to be a heterogeneous disease, where diverse disease courses culminate in a common end point [6,7]. Up to now, samples of hip and knee OA are often pooled in experimental studies, assuming that both share the same pathophysiology. However, clinical and epidemiological observations suggest that hip and knee OA differ with respect to disease onset, age distribution, and severity of clinical presentation. We hypothesized that the heterogeneous presentation of OA is a result of a heterogeneous underlying pathophysiology. The purpose of this study was to examine the mononuclear cell infiltration and cytokine release profile in the synovial membrane of hip and knee OA in order to search for potential differences in inflammatory pathways. A growing body of evidence has shown that inflammatory pathways significantly contribute to OA pathophysiology [8,9]. Inhibition of these inflammatory pathways can slow OA progression in animal models, which makes inflammation a promising target for OA treatment [10,11]. In order to stratify OA pathophysiology, we aimed to analyze if hip and knee OA, which show differences in severity and duration of symptoms as well as disease progression, also differ in their immunological pattern [12].

Ultimately, defining the pathophysiology of OA subtypes and characterizing common but also different pathways could prove helpful in understanding disease pathophysiology and developing specialized osteoarthritis disease-modifying interventions.

## 2. Methods

### 2.1. Study Population

**Figure 1:**
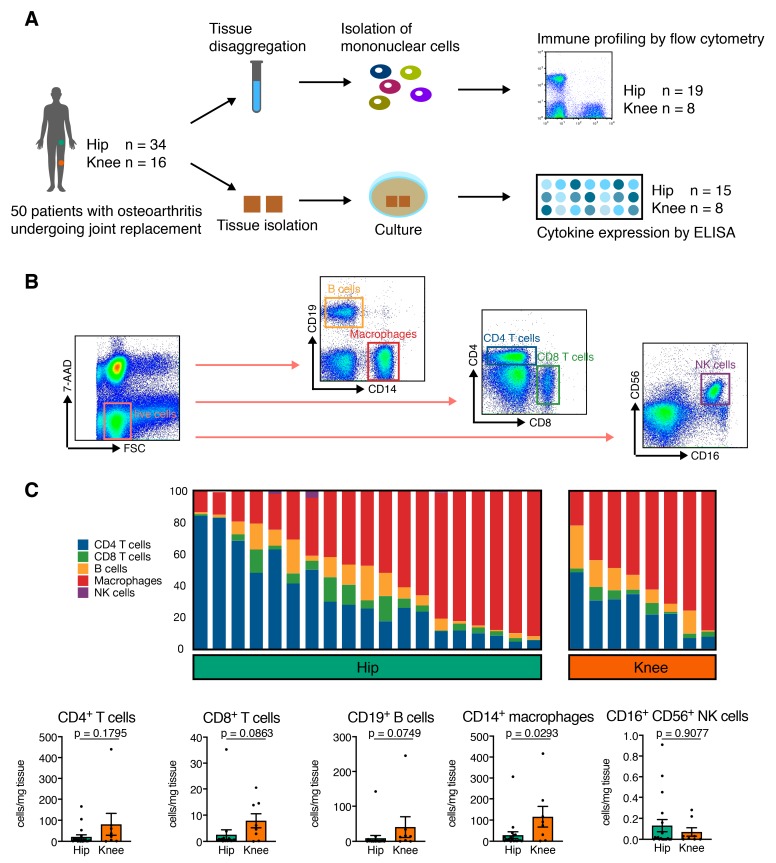
Harvesting of synovial membrane and flow cytometry and cytokine expression analysis of synovial membrane from patients with different types of osteoarthritis. (**A**) Synovial tissue was obtained from 50 patients with hip (*n* = 34) and knee (*n* = 16) osteoarthritis, disaggregated, and digested. Following fresh isolation of mononuclear cells, *n* = 19 hip and *n* = 8 knee samples were analyzed by flow cytometry to profile the contribution of different lineages. Cells from *n* = 15 hip and *n* = 8 knee OA samples were cultured for 24h, and quantitative release of 48 canonical cytokines was assessed using multiplex ELISA. (**B**) Schematic gating strategy to identify CD4+ T cells, CD8+ T cells, CD19+ B cells, CD14+ macrophages, and CD16+ CD56+ NK cells from disaggregated osteoarthritic synovial membrane. (**C**) Relative contribution of different mononuclear cell lineages to the synovial membrane infiltrate suggests a spectrum of mononuclear infiltration ranging between two poles, characterized by a predominantly CD4 T cell infiltrate and a predominantly macrophage infiltrate. (**D**) CD14+ macrophages and CD8+ T cells are expanded 3.8-fold in synovial membrane of knee OA compared to hip OA (*p* = 0.0293 and *p* = 0.0863 by Mann–Whitney test).

50 patients with OA (34 patients with hip OA and 16 patients with knee OA) were included in this study. Due to methodological setup, one part of the study population was utilized for flow cytometry analysis and the other part for multiplex analysis of cytokine expression (Figure 1A). The study population characteristics are summarized in Table 1. We determined osteoarthritis based on patient history, clinical examination, and radiographic evaluation according to the criteria defined by the American College of Rheumatology. To assess the radiographic severity of OA, we used the Kellgren and Lawrence (K&L) scoring system. All patients had osteoarthritis with Kellgren–Lawrence (K&L) scores III or IV, and received total hip or total knee replacement surgery at Heidelberg University Hospital. The diagnosis was re-evaluated intra-operatively. None of the patients had an underlying inflammatory pathology or signs of systemic inflammation, as determined by blood analysis. None of the patients were treated with corticosteroids, disease-modifying anti-rheumatic drugs, or biologics, which were considered exclusion criteria. The local ethics committee of the University of Heidelberg approved the study (approval code: S333/2007) and all patients provided written informed consent prior to study enrollment.

### 2.2. Sample Preparation for Flow Cytometry Analysis

Prior to surgery, peripheral blood samples were obtained and collected in EDTA-containing tubes. Synovial membrane (SM) samples were harvested from the resected capsule of the hip and the suprapatellar pouch of the knee during surgery and prepared as previously described [13]. SM samples were rinsed twice with phosphate buffered saline (PBS), minced finely with sterilized scissors, and digested with collagenase B (1 mg/mL; Roche, Basel, Switzerland) and bovine testicular hyaluronidase IV (2 mg/mL; Sigma-Aldrich, St. Louis, Missouri, USA) at 37 °C for 2 h in RPMI 1640 culture medium (Invitrogen, Carlsbad, California, USA) supplemented with 10 μg/mL penicillin-streptomycin (Invitrogen, USA) and 5% FCS (Biochrom AG, Berlin, Germany). The cell suspension was filtered through a 100 μm (BD Biosciences, Franklin Lakes, New Jersey, USA) and a 40 μm pore-size cell strainer (EMD Millipore, Burlington, Massachusetts, USA) to remove any undigested tissue. The filtered cell suspension was washed twice with PBS. Mononuclear cells were isolated from EDTA anti-coagulated whole blood and SM cell suspensions using Ficoll-PaqueTM PLUS (GE Healthcare, Chicago, Illinois, USA) density gradient centrifugation. To identify mononuclear cells according to their cell surface markers, multi-color flow cytometry was used. Briefly, mononuclear cells were washed twice in magnetic affinity cell sorting staining buffer (MACS; Miltenyi Biotec, Cologne Germany), blocked with FCS blocking reagent, and then stained (30 min at 4 °C) with the following monoclonal antibodies (mAb): CD4-allophycocyanin (APC)-cyanin 7 (Cy7) (BD clone: RPA-T4), CD8-VioBlue (Miltenyi clone: BW135/80), CD14-fluorescein isothiocyanate (FITC) (BD Pharmingen clone: M5E2), CD16-phycoerythrin (PE)-Cy7 (BD clone: 3G8), CD19-PE (Miltenyi clone: LT19), and CD56-APC (Miltenyi clone: AF12-7H3). The cells were washed again and taken into a final volume of 200 μL MACS staining buffer. Immediately before flow cytometric detection, cells were stained with 7-aminoactinomycin D (7-AAD; eBioscience, Santa Clara, California, USA) with a final concentration of 0.5 μg/mL. A total of 10^5^ events were assessed and analyzed with a MACS-Quant flow cytometer (Miltenyi Biotec, Cologne Germany). Data analysis was performed using FlowJo version 9.8.1 (TreeStar Inc., Ashland, Oregon, USA). A representative gating strategy is shown in Figure 1B. Cell debris and dead cells were excluded (7-AAD staining and forward-scatter profile) and mononuclear cells were gated based on their forward- and side-scatter profiles. Mononuclear cell subsets were defined by their surface marker expression as CD4^+^ T cells, CD8^+^ T cells, CD14^+^ macrophages, CD19^+^ B cells, and CD16^+^CD56^+^ natural killer (NK) cells. The cut-off for all cell surface markers was defined based on isotype controls.

### 2.3. Multiplex Cytokine Analysis from Synovial Membrane Supernatants

Synovial membrane samples were harvested from hip and knee OA joints as described above. SM samples were rinsed twice with phosphate buffered saline (PBS), minced finely with sterilized scissors, and taken into culture for 24 h. The supernatants were harvested and stored at −80 °C. The Pro-Human Cytokine Multiplex Assays (Bio-Rad, Germany) was used to analyze the cytokines in synovial fluid samples. The 27-plex analyses for Eotaxin (CCL11), FGF-Basic, G-CSF, GM-CSF, interferon (INF)-γ, IL-1β, IL-1ra, IL-2, IL-4, IL-5, IL-6, IL-7, IL-8 IL-9, IL-10, IL-12-p70, IL-13, IL-15, IL-17, IP-10, monocyte chemotactic protein 1 (MCP-1, CCL2), macrophage inflammatory protein-1α (MIP-1α, CCL3), MIP-1β (CCL4), PDGF–bb, regulated upon activation normal T cell expressed and activated (RANTES, CCL5), TNF-α, and vascular endothelial growth factor (VEGF). The 21-plex contains CCL27, GRO-α, HGF, IFN-α2, IL-1α, IL-2Rα, IL-3, IL-12p40, IL-16, IL-18, leukemia inhibitory factor (LIF), MIF, MCP-3 (CCL-7), M-CSF, MIG (CXCL9), β-NGF, stem cell factor (SCF), SCGF-β, SDF1α (CXCL-12), TNF-β, and TRAIL. Multiplex assays were carried out according to the manufacturers’ instructions and run on the Luminex 200 platform. Bio-Plex Manager version 5.0 was used for data processing. Cytokine and chemokine concentrations were calculated by reference to the standard curve. The sensitivity of the multiplex kit was <5 pg/mL.

### 2.4. Cytokine Expression Analysis

Following ELISA measurements, we used a two-step computational pipeline to test the association between different samples. First, to allow simultaneous analysis of different soluble mediators with concentration profiles that differ in several orders of magnitude, the expression value of each soluble mediator was transformed: Values above the detection threshold (*n* = 3 of 1012 measurements) were substituted by the highest measured value for the respective soluble mediator. Values below the detection threshold (*n* = 17 of 1012 measurements) were substituted by zero. Then, expression values were standardized between 0 and 1. Soluble mediators for which less than 25% of measurements were usable were excluded (HGF, MIF, IL-6, IL-8), retaining 44 cytokines for downstream analysis. From the resulting matrix, we calculated the Pearson correlation between all samples and the spearman correlation between all soluble mediators. In order to test if cytokines were differentially expressed between samples from hip and knee OA, we used the calculated mean and standard deviation for each cytokine.

### 2.5. Statistical Analysis

Demographic parameters between study groups were compared using the unpaired *t*-test for parametric data, the Mann–Whitney test for non-parametric data, and the Fischer’s exact test for proportions. A Kolmogorov–Smirnov test was performed to test the variables for normal distribution. The Mann–Whitney test was used for analysis of mononuclear cell frequencies between the two study groups. The unpaired *t*-test was performed to assess the differences between cytokine expression in SM samples due to the predominantly Gaussian distribution. Correlation analysis between radiographic scores and cytokines was done by Spearman’s correlation analysis for non-parametric data and Pearson’s correlation analysis for parametric data. All reported *p*-values are two-tailed. A *p*-value <0.05 was considered to show a statistically significant difference. Statistical analysis was performed using Prism version 5 software (GraphPad Software, Inc., San Diego, California, USA), SPSS version 22·0 (IBM, Armonk, New York, USA), and R version 3.5.1.

## 3. Results

### 3.1. Description of the Study Population

The two study groups showed significant differences regarding the demographic parameters age at surgery and BMI **(**Table 1**)**: Hip OA patients were significantly younger (hip: 63.2 ± 12.2 years vs. knee: 71.9 ± 5.6 years; *p* = 0.001), while the average BMI was significantly higher in knee OA (hip: 26.0 ± 4.6 kg/m^2^ vs. knee: 29.7 ± 6.2 kg/m^2^ *p* = 0.022). The laboratory parameters C-reactive protein (CRP) and leucocytes were within standard range, and thus did not suggest any signs of systemic inflammation at the time of surgery. Our first approach was to analyze peripheral blood of both patient groups for frequency of mononuclear cell subsets (Figure 1A,B). There was no significant numeric difference for mononuclear cells in peripheral blood (knee OA: 88.6 ± 6.5 cells/mg; hip OA: 90.6 ± 5.1 cells/mg, *p* = 0.4431), CD14^+^ macrophages (knee OA: 28.1 ± 12.8 cells/mg; hip OA: 31.3 ± 9.4 cells/mg, *p* = 0.5404), CD8^+^ cytotoxic T cells (knee OA: 13 ± 6.8 cells/mg; hip OA: 13.5 ± 4.6 cells/mg, *p* = 0.8394), CD4^+^ T helper cells (knee OA: 25.6 ± 11.7 cells/mg; hip OA: 31.7 ± 6.7 cells/mg, *p* = 0.1508), CD19^+^ B cells (knee OA: 5.8 ± 1.2 cells/mg; hip OA: 7.5 ± 4.3 cells/mg, *p* = 0.279), and CD16^+^CD56^+^ natural killer cells (knee OA: 7.6 ± 5.4 cells/mg; hip OA: 7 ± 2.5 cells/mg, *p* = 0.7010).

Next, in the absence of systemic inflammatory differences, we proceeded to directly test the site of joint destruction: the synovial membrane.

### 3.2. Hip OA Synovial Membrane Displays Higher Amounts of Mononuclear Cell Infiltration

Mononuclear cells from SM of the affected joints were analyzed by flow cytometry for surface marker expression and frequencies (Figure 1B,C and Table 2). The amount of total mononuclear cells was higher in knee than hip OA (knee OA: 827.4 ± 254.1 cells/mg; hip OA: 213.9 ± 118,3 cells/mg, *p* = 0.0662). Only very few CD16^+^CD56^+^ NK cells were detected in the SM of both groups **(**Figure 1C). Interestingly, the contribution of CD14^+^ macrophages and CD4^+^ T helper cells varied greatly between individual donors, defining two distinct poles characterized by a predominantly CD4^+^ T cell infiltrate versus a predominantly CD14^+^ macrophage infiltrate **(**Figure 1C). This was seen in both hip and knee samples, highlighting the very individual nature of a disease course. When considering the total numbers of cells, CD14^+^ macrophages (knee OA: 116.1 ± 48.42 cells/mg; hip OA: 29.45 ± 15.88 cells/mg, *p* = 0.0293) were significantly increased in knee OA, and CD8^+^ cytotoxic T cells showed a trend for increase in knee OA (knee OA: 7.88 ± 2.67 cells/mg; hip OA: 2.59 ± 1.83 cells/mg, *p* = 0.0863) **(**Figure 1D), suggesting that hip and knee osteoarthritis are distinct with regard to the inflammatory processes that take place in the synovial membrane.

### 3.3. Cytokine Expression Profiles Distinguish Hip and Knee Osteoarthritis

**Figure 2:**
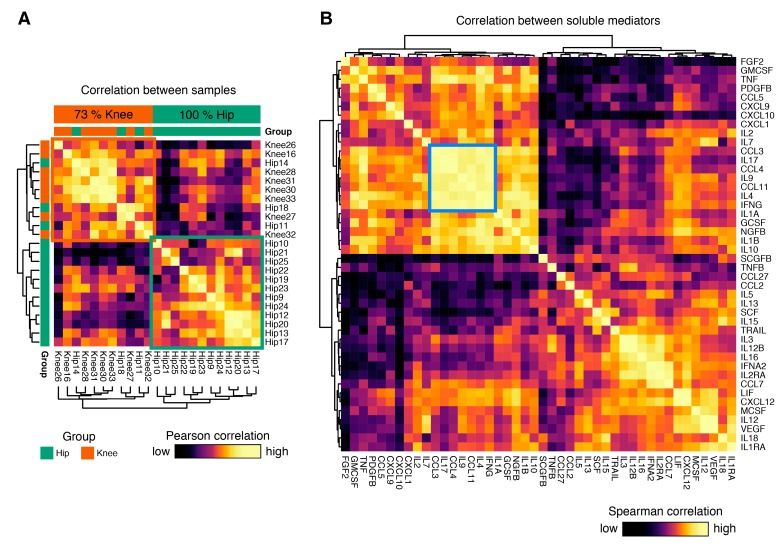
Visualization of the correlation matrix between samples and soluble mediators. (**A**) Heatmap showing the clustered Pearson correlation between all sample-sample pairs based on expression of all cytokines. This unsupervised approach revealed two major groups of samples, one exclusively consisting of hip OA samples (12/12, 100%), and one predominantly of knee OA samples (8/11, 73%). (**B**) Heatmap showing the spearman rank correlation between all cytokine-cytokine pairs. One highly correlated cluster of cytokines consisting of IFNG, IL4, CCL11, IL9, CCL4, IL17, and CCL3 was identified.

In the next step, we cultured unmanipulated synovial membrane samples and analyzed their cytokine release profile in the supernatant by multiplex ELISA analysis. Standardized cytokine expression values were used to calculate pairwise Pearson correlation coefficients between all individual samples. Hierarchical clustering of these sample to sample distances revealed a striking separation, predominantly driven by the joint involved in osteoarthritis (Figure 2A): Two major clusters were identified. One exclusively consisted of hip OA samples (12/12, 100%), and the second was predominantly formed by knee OA samples (8/11, 73%). This finding, obtained in an unsupervised approach, suggests that isolates from hip and knee OA display fundamentally distinct cytokine release profiles.

### 3.4. Hip OA Presents a Higher Inflammatory Cytokine Expression Profile

**Figure 3:**
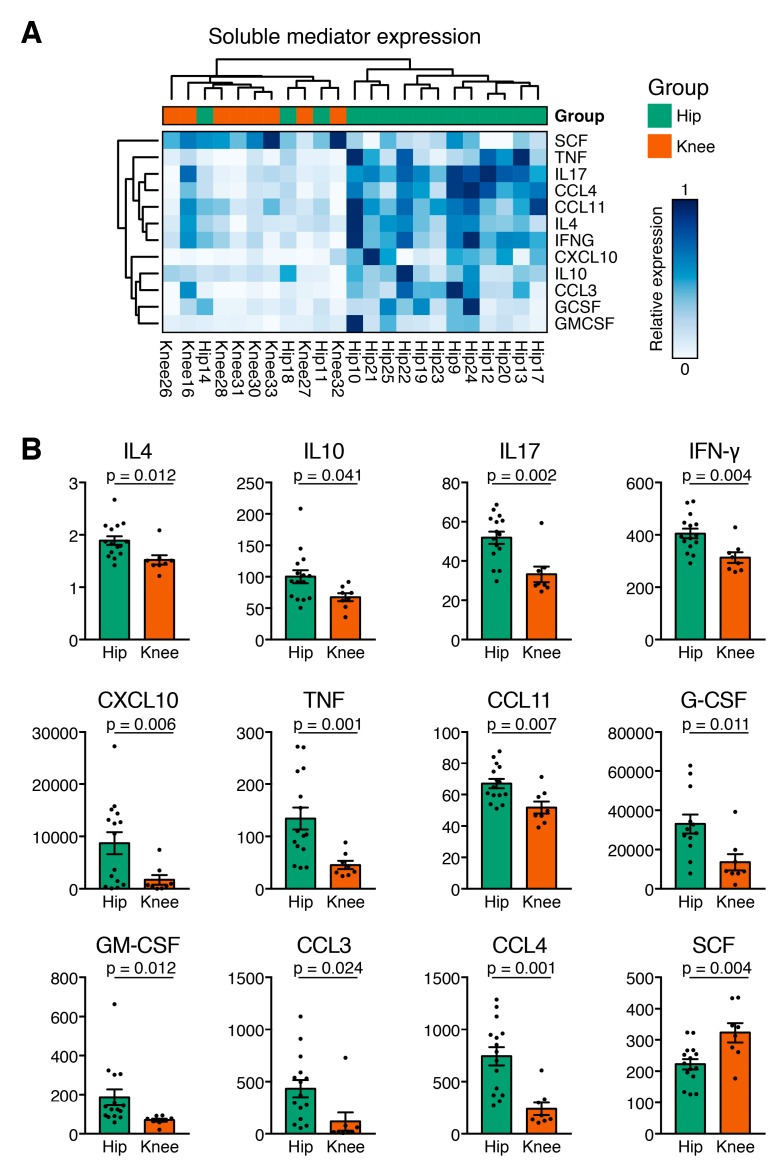
Cytokine expression profiles in synovial membrane mononuclear cells distinguish hip and knee osteoarthritis. (**A**) Heatmap visualizing relative expression values of statistically significantly, differentially released cytokines between hip and knee OA. Rows and genes were clustered in an unsupervised manner. Samples clustered in a similar fashion to Figure 2A and relative expression revealed two distinct soluble mediator programs, suggesting coordinated cytokine programs. (**B**) Dot plots of the 12 cytokines that are overexpressed in one subtype of OA compared to the other. The unpaired *t*-test was performed to assess the differences between end-stage hip and knee osteoarthritis (OA) SM samples due to the predominantly Gaussian distribution. For cytokines which did not show Gaussian distribution, a Mann–Whitney U-test was performed. All reported *p*-values are two-tailed. A *p*-value < 0.05 was considered to show a statistically significant difference.

We further examined the pairwise Spearman correlation between individual soluble mediators. While both positive and negative correlations between soluble mediators were found, a cluster of strongly correlated cytokines consisting of CCL3, IL-17, CCL4, IL-9, CCL11, IL-4, and IFN-γ stood out (Figure 2B). In hip OA, SCGF-β, and G-CSF, and in knee OA SCGF-β and HGF showed the highest concentration, followed in hip OA by HGF, MIG–CXCL9, IP–10, VEGF, GRO-α, and LIF, and in knee OA by G–CSF, MIG–CXCL9, VEGF, GRO-α, MCP–1–MCAF, and LIF. Hip and knee OA samples differed significantly in cytokine expression (Figure 3A). Of the 44 measured cytokines, 11 were present at significantly higher levels in hip OA compared to knee OA: Eotaxin, G-CSF, GM-CSF, IFN-γ, IL-4, IL-10, IL–17, IP–10, MIP–1α/CCL3, MIP–1β/CCL4, and TNF-α (Figure 3A). Eight of those 11 mediators possess pro-inflammatory characteristics (Eotaxin, GM-CSF, IFN-γ, IL–17, IP–10, MIP–1α/CCL3, MIP–1β/CCL4, TNF-α), while three anti-inflammatory cytokines (G-CSF, IL-4, IL-10) were increased significantly in hip OA compared to knee OA **(**Figure 3A). Interestingly, knee OA synovial tissue isolates released 78.5% more SCF than cells from hip OA (Figure 3B). Other soluble mediators showed comparable levels between the groups. Of note, the pro-inflammatory cytokine IL-6 was highly expressed in all analyzed samples and surpassed the upper detection threshold in all but one of the analyzed samples (see methods). To rule out the possibility that cytokine expression was confounded by BMI or age of the study participant, we applied a multivariable linear regression model to correlate immune cell infiltration and cytokine expression with age and BMI as potential confounding variables in both groups. Age and BMI were not related to the differentially expressed cytokines between hip and knee OA. 

As both the hip and knee OA group contained patients with radiographic Kellgren and Lawrence scores III and IV, we additionally examined the data separately for radiographic scores. In the cytokine release data, all samples from knee OA were scored as K&L III. In hip OA, 11 samples were from K&L III, and 4 samples had K&L IV. No cytokines were differentially expressed between K&L score III hip OA and K&L score III knee OA, suggesting that this asymmetry did not influence our initial results. Direct comparison of cytokine expression from K&L III in hip OA vs. K&L III in knee OA identified significantly higher production of IL-17, GM-CSF, CXCL10, CCL3, CCL4, PDGF-BB, and TNF in the Hip OA cohort, and higher expression of SCF, SCGF-beta, and IL-13 in the knee OA cohort (Figure S1). Of these cytokines, the expression of GM-CSF, CXCL10, CCL3, CCL4, TNF, and SCF overlapped with the analysis independent of K&L scores, thereby demonstrating a core cytokine signature between hip and knee OA independent of the K&L score.

### 3.5. Identification of Coordinated Cytokine Expression Patterns in Hip and Knee Osteoarthritis

**Figure 4:**
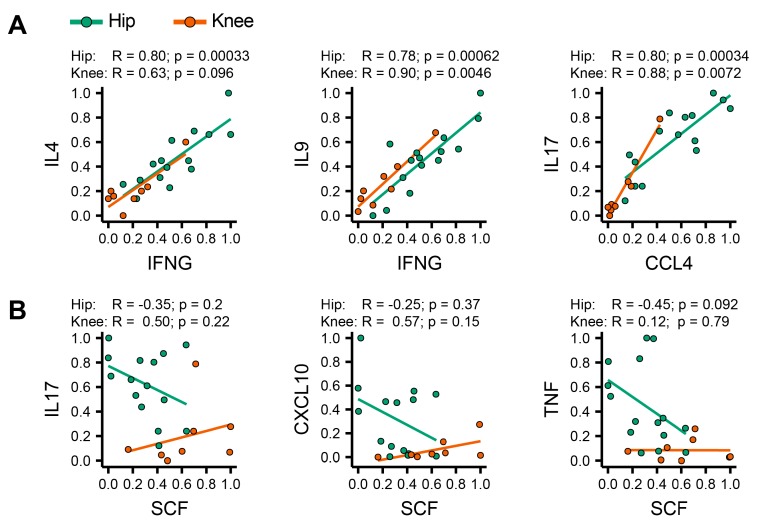
Correlation between soluble mediators. (**A**) We selected pairs of highly correlated cytokines from the cluster shown in Figure 2B and plotted individual relative expression values against each other. IFNG, IL4, IL9, CCL4, IL17, CCL11 (not shown), and CCL3 (not shown) are each highly predictive of each other’s expression. (**B**) Context sensitivity of coordinated cytokine expression programs. Expression of stem cell factor (SCF) is not correlated with TNF and slightly positively correlated with IL-17 and CXCL10 in knee OA, but strongly negatively correlated with all three cytokines in hip OA. Spearman correlation values and p-values are shown separately for hip and knee OA.

Consistent with differences in mononuclear cell infiltration into the joints, we found differences in the cytokine release profiles between hip and knee OA isolates. These differences in cytokine release appeared to be coordinated, as we identified highly correlated expression pairs, such as IFN-γ–IL-4, IFN-γ–IL-9 and CCL4–IL-17 that were positively correlated in both hip and knee OA. (Figure 4A). However, SCF, which was observed in higher quantities in knee OA samples, correlated poorly with IL-17, CXCL-10, and TNF expression in knee, but showed a strong negative correlation with these cytokines in hip OA (Figure 4B). These co-variances in cytokine expression appear highly coordinated, suggesting a distinct role in the pathophysiology of these two diseases. In summary, our results reveal a high degree of heterogeneity between individual OA patients. We were able to show that both mononuclear cell infiltration patterns and cytokine release profiles differ significantly between hip and knee OA, adding evidence that these two disease manifestations possess distinct features. 

## 4. Discussion

Osteoarthritis is increasingly recognized as a heterogeneous disease [12,14]. In daily practice, clinical, demographic, and radiographic data are used to subdivide OA into primary versus secondary, or early versus late OA. Even though the granularity of this schema is low and the classification is imprecise since a vast amount of information is missed in the clinical setting, this schema provides a first approach in understanding OA as a heterogenetic disease. Integration of these clinical data in the interpretation of cellular and molecular analysis in OA pathophysiology may represent the key to developing disease-modifying medication for subsets that require specialized interventions.

However, up to this point osteoarthritis of hip and knee have been viewed as a manifestation of the same disease in different joints, and many studies on OA pool samples from different joints for analysis. If OA from different joints had distinct cellular and molecular properties, then attempts to identify disease biomarkers or prognostic parameters could potentially miss discovery power in a pooled analysis [14]. Therefore, we investigated the immune cell infiltration pattern and cytokine release profiles of hip and knee osteoarthritis.

Healthy synovial membrane is an acellular structure assembled by an intimal lining layer and a synovial sublining layer. Infiltration of immune cells, structural changes such as stromal edema, and proliferation of blood vessels accelerate the loss of the SM-lining structure [15]. Especially macrophages and T cells seem to be the predominant cells in OA synovium [16]. By utilizing flow cytometry to analyze the number of mononuclear cells in the SM, our study shows a previously unappreciated difference in the inflammatory pattern of hip OA and knee OA synovium: Total mononuclear cells in the synovial tissue were increased fourfold in knee OA compared to hip OA. Further characterization of this mononuclear infiltrate showed that CD14^+^ macrophages were the largest cell population in hip OA and knee OA, accompanied by CD4^+^ T cells, CD19^+^ B cells, CD8^+^ T cells, and CD16^+^CD56^+^ NK cells in descending order.

Previous immunohistochemical studies of osteoarthritic joints have shown that T cells are detectable in synovial tissue at an early disease stage [17]. Using the same immunohistochemical techniques, the sublining layer was shown to contain predominantly CD4^+^ and CD8^+^ T cells, and the intimal layer predominantly CD4^+^ T cells. Our study is in accordance with previous publications, which showed predominant CD4^+^ and CD8^+^ mononuclear cell infiltration in mid to severe OA [18]. These findings imply that T cells play a crucial role in patients with OA [19]. Especially in earlier OA stages, CD4^+^ T cells are described to induce synovitis [20]. Our study complements those results by specifying the different mononuclear cell populations with their frequency in OA synovial tissue and reveal different composition of inflammatory cells in different osteoarthritic joints.

As first part of active immune response, activated resident macrophages represent the major cell population, followed by T-lymphocytes and plasma cells, which immigrate in the affected joints where they play a relevant role in initiation of OA and induce subsequently pathologic elevation of inflammatory cytokines [13,15,21]. It is known from rheumatoid arthritis (RA) that macrophages and T cells interact closely to drive chronic inflammation [22]. In accordance with our data, activated CD14^+^ macrophages appear to mediate structural progression and pain in knee OA by playing a central role in maintenance of inflammation [23]. Our results show a higher mononuclear cell infiltration in knee OA compared to hip OA. We suspect macrophages to be the first cell line in initiation of OA with consecutive production of cytokines and hypothesize the higher amount of mononuclear cells and lower amount of cytokines in knee OA compared to hip OA could be caused due to the faster and more severe progress in hip OA, with declining amount of cells and consequent rising of cytokines as soluble stimuli parallel to advanced joint destruction. Considering hip OA as the more severe and faster worsening OA manifestation, the decrease of cell infiltration could indicate a later time point in disease progression with contribution of synovial degradation.

Taken together, CD14+ macrophages and CD4+ T cells appear to mediate much of OA immunopathology. Thus, functional investigations of macrophage and T cells and their interaction within synovial tissues could be of utmost relevance for the understanding of pathological events in OA. In addition to immune cells, fibroblasts are critical effector cells in arthritis. Recent studies have described distinct subsets of fibroblasts based on different surface protein expression, transcriptome, and functional properties, including inflammatory cytokine release. [24,25,26] In particular, a subset of FAPα+ THY1+ fibroblasts expanded in osteoarthritis seems capable of releasing larger quantities of the matrix metalloproteinases MMP3, MMP9, and MMP13, which are known catabolic enzymes in OA.

Besides the immune cell infiltration, an inflammatory cytokine environment is present in OA joints. We have previously shown that in end-stage OA, a shift towards a pro-inflammatory cytokine environment takes place [27]. A number of studies document that TNF-α, IL-1α, and IL-1β act as the main pro-inflammatory cytokines, and are thought to play a crucial role in the initiation and progression of OA [28,29]. A number of studies suggest a significant increase of IL-6, IL-8, IL-12, IL-17, and IL-18 as pro-inflammatory players in OA [30,31]. Especially IL-6 is thought to play a significant role in early OA and also seems to increase with a higher grade of obesity (BMI > 30), which applies only partly to our patient cohort. IL-1α and IL-1β were present at high concentrations in our study, which could suggest a more relevant role of these cytokines in later OA stages. Further cytokines such as IFN-γ, SCF, and Eotaxin have been described in OA joints [4,31,32,33].

In direct comparison between different joints, our study reveals that hip OA is affected by significantly higher cytokine levels in comparison to knee OA, with a marked shift towards a pro-inflammatory milieu including IFN-γ, TNF-α, and IL-17. IFN-γ is produced by a broad variety of cells like NK and T-cells, is an important activator of macrophages, and has an immunostimulatory and immunomodulatory character. The significantly higher presence of IL-17 in hip OA could suggest an important role in hip OA pathophysiology, as it is able to induce inflammatory cytokines such as TNF-α, IL1β, and IL-6 to amplify joint destruction [21,34,35]. TNF-α plays a key role in the pathogenesis of OA by stimulating proteases and PGE2 to induce cytokine cascades through synovial cells and chondrocytes, leading to accelerated degradation of the synovial membrane and articular damage [29,36].

Interestingly, the two anti-inflammatory cytokines IL-4 and IL-10 showed a higher concentration in hip OA than knee OA. IL-4 and IL-10 stimulate proliferation and differentiation of T and B cells, and are associated with a chondroprotective effect. Both cytokines inhibit secretion of IL1β, TNF-α, MMPs and can thereby suppress inflammation of the synovial membrane and reduce variations of proteoglycans which maintain OA pathophysiology [29,37].

Growth factors like SCGF-β, G-CSF, HGF, and VEGF showed the highest absolute concentrations in both disease groups. First, they could play an important role in progression of OA by promoting abnormal osteoblast expression from the subchondral bone plate and inducing subchondral bone sclerosis [38]. Second, the increased level of the pro-angiogenetic VEGF induces vascularization and blood flow in the synovial tissue and may maintain synovial hypertrophy and mononuclear infiltration with additional inflammatory cytokine release [39,40]. Finally, aberrated growth factor homeostasis in osteoarthritic joints could lead to disease progression due to the imbalance of consequential cytokine expression [41].

Regarding MIG–CXCL9, other studies suggest a higher concentration in OA by elevating expression of pro-inflammatory cytokines and suppression of chondrocyte multiplication [42]. IP-10 (CXCL10) as a chemokine produced by leukocytes and synovial tissue cells plays a substantial aspect in the conglomeration of inflammatory cells and immune response at inflammatory occurrence. In inflammatory arthritis, the IP-10/CXCR3-axis mediates chemotaxis of T cells to inflamed synovium and promotes joint damage [43]. In our analysis, IP-10 was highly abundant in both OA joints, but with significantly higher levels in hip OA. This is in accordance to previous studies which have reported high IP-10 levels, together with MDC, in end stage knee OA [44]. The higher abundance of IP-10 in hip OA than in knee OA or healthy subjects was confirmed by immunohistochemistry, which supports our findings [14]. Notably, the same study found that IP-10, as well as IL-6 and MDC, correlated well with pain in hip OA. SCF was the only cytokine significantly increased in knee OA. Apart from its role in hematopoiesis, it is released from precursors and mature mast cells (MC) and induces their development, chemotaxis, activation, and adhesion. Studies suggest that SCF is responsible for hyperplasia and inflammation in arthritis [45].

Besides the above-mentioned differences in cytokine release patterns between both OA groups, a broad variety of pro-inflammatory mediators such as LIF, CCL27, IL-1a, and IL-12-p70 was present in the affected joints without a significant difference between groups.

In summary, were able to show significantly higher levels of many inflammatory mediators in isolates from hip OA which potentially accelerate disease progression. Thus, different affected joints in OA may differ in inflammatory pathophysiology.

In our study, hip OA and knee OA displayed significant differences regarding BMI and age at surgery, and these differences have implications for data interpretation: Obesity is one of the strongest risk factors of OA, with a higher correlation especially in knee OA. Beside the increased loading stress in joints, obesity leads to metabolic abnormalities and systemic inflammation [46]. Thus, the significantly higher infiltration of the SM with mononuclear cells in knee OA could potentially be associated with the higher BMI in this group. Apart from BMI, cytokine levels are influenced by OA severity and demographic factors including age and gender [47,48]. Further, it has been shown that older patients with OA seem to have a higher percentage of T cells in their synovial tissue than younger affected OA patients [49]. Considering this, the higher CD8^+^ T cell infiltration in knee OA could also be affected by the higher age of this group. Therefore, confounding was excluded by use of a multivariable linear regression model.

The detected demographic heterogeneity between hip and knee OA with respect to age at time of surgical intervention and BMI is additional evidence for differences in disease pathophysiology between OA joints. This idea is further supported by clinical observations: Patients with manifested hip OA suffer more severe symptoms at an earlier age and require total joint arthroplasty after a shorter disease course. In contrast, knee OA manifests at later age with less severe symptoms and a longer period until arthroplasty. Our study supports these differences in age at surgery and BMI between both disease groups, which are both higher in knee OA. Although we did not observe differences in K&L score, more severe radiographic changes were displayed in hip OA, which undergirds hip OA as a more severe and faster declining condition, as supported by previous studies [12]. The results obtained in our and previous studies open the possibility that other joints affected by OA such as hands, the spine, or the shoulder may also be distinct. Indeed, it was already demonstrated that articular cartilage in the ankle compared to the knee has a higher cellularity [50]. Consistent with this, proteoglycan and collagen synthesis was higher in chondrocytes from ankle compared to knee in vitro, potentially offering an explanation for the faster progression of OA in knees compared to ankles [50].

We acknowledge that our study has limitations. By focusing on the comparison between hip and knee OA using tissue obtained during joint arthroplasty, this study did not include healthy controls. The inclusion of healthy synovial tissue may be possible in the future through the increasing availability of synovial tissue microbiopsies. Furthermore, by only including patients with primary OA, our study excluded secondary factors that also predispose joints to OA. Also, patients with polyarticular osteoarthritis were not included because of the vague definition in the literature and the potentially multimodal causes which lead to a generalized osteoarthritis [51]. By culturing whole synovial membrane tissue, our study is not able to resolve cell type specific release of cytokines. Finally, although hip and knee OA separated well in our analysis, it is unclear into how many disease subsets OA can be divided or if osteoarthritis is better modeled through a continuous disease process.

## 5. Conclusions

Taken together, this study establishes hip and knee OA as distinct disease subsets with respect to the immune cell infiltration pattern in the synovium and cytokine release profiles. Exploration of immune cell infiltration and inflammatory pathways is highly relevant to understand disease pathophysiology. This study represents an early step in deciphering OA heterogeneity, and sets the stage for subsequent, functional studies on the role of inflammatory biomarkers and mononuclear cells in OA, which are required to assess disease progression. This and potentially other stratification strategies may inform clinical decision making in the future and pave the way for personalized therapies in osteoarthritis.

## Figures and Tables

**Table 1:** Study population.

	Total Study Population	Hip OA	Knee OA	*p*-Value
Number of patients (*n*)	50	34	16	
Gender Male/Female	M = 27 (54.0%) F = 23 (46.0%)	M = 18 (52.9%) F = 16 (47.1%)	M = 9 (56.2%) F = 7 (43.8%)	*n*.s.
Age at surgery (Years) Mean ± SD (range)	66.0 ± 11.3 (38–88)	63.2 ± 12.2 (38–88)	71.9 ± 5.6 (62–83)	0.0001 *
Operation side (*n*) (%) Right/Left	R = 29 (58.0%) L = 21 (42.0%)	R = 20 (58.8%) L = 14 (41.2%)	R = 9 (56.2%) L = 7 (43.8%)	*n*.s.
BMI (kg/m^2^) mean ± SD (range)	27.2 ± 5.4 (16.9–43.2)	26.0 ± 4.6 (16.9–36.0)	29.7 ± 6.2 (19.8–43.2)	0.022 *
Leukocytes (cells/nl) mean ± SD (range)	7.0 ± 1.6 (4.1–10.1)	7.1 ± 1.7 (4.1–10.1)	6.8 ± 1.3 (5.0–10.1)	*n*.s.
C-reactive protein (mg/l) mean ± SD (range)	3.6 ± 3.7 (2.0–17.8)	3.5 ± 3.7 (2.0–17.8)	3.8 ± 3.9 (2.0–16.5)	*n*.s.
ESR (mm/h) mean ± SD (range)	13.6 ± 12.4 (1–77)	13.4 ± 13.6 (1–77)	14.0 ± 9.0 (2–30)	*n*.s.
K&L Score (*n*) (%) 3/4	K&L 3 = 36 (72.0%) K&L 4 = 14 (28.0%)	K&L 3 = 22 (64.7%) K&L 4 = 12 (25.3%)	K&L 3 = 14 (87.5%) K&L 4 = 2 (12.5%)	*n*.s.

Demographic and clinical parameters of the study population are shown. Values are given as mean ± SD (range) for parametric data and as proportions for categorical data. Statistical analysis of demographic parameters between study groups was performed using the unpaired *t*-test for parametric data and the Fischer’s exact test for proportions. All reported *p*-values are two-tailed. A *p*-value <0.05 was considered to show a statistically significant difference. BMI = body mass index; ESR = Erythrocyte Sedimentation Rate; K&L Score = Kellgren Lawrence Score; *n*.s. = not significant; * *p* < 0.05.

**Table 2:** Frequencies of mononuclear cells in synovial membrane of end-stage hip and knee osteoarthritis patients.

	Hip OA	Knee OA	*p*-Value
Number of patients (*n*)	19	8	
Sample volume (g)	1.75 ± 0.17 (0.83–3.62)	1.46 ± 0.64 (1.23–1.78)	0.5755
Total mononuclear cells (cells/mg)	213.9 ± 118,3 (10.43–2321)	827.4 ± 254.1 (11.55–1998)	0,0662
CD14+ macrophages (% of mononuclear cells)	16.83 ± 3.55 (1.53–46.17)	14.89 ± 3.39 (4.57–30.40)	0.6878
CD14+ macrophages (cells/mg)	29.45 ± 15.88 (0.83–306.4)	116.1 ± 48.42 (1.75–414.6)	0.0293 *
CD4+ T cells (% of mononuclear cells)	10.63 ± 3.11 (0.52–51.30)	7.79 ± 2.47 (0.79–22.00)	0.8966
CD4+ T cells (cells/mg)	21.37 ± 9.9 (0 – 165)	80.88 ± 52.25 (1–440)	0.1795
CD8+ T cells (% of mononuclear cells)	1.15 ± 0.21 (0.19–3.70)	1.08 ± 0.33 (0.34–2.82)	0.8966
CD8+ T cells (cells/mg)	2.59 ± 1.83 (0.16–35.13)	7.88 ± 2.67 (0.07–20.52)	0.0863
CD19+ B cells (% of mononuclear cells)	1.74 ± 0.41 (0.42–6.13)	3.58 ± 1.43 (0.2–12.30)	0.2332
CD19+ B cells (cells/mg)	9.14 ± 7.43 (0.05–142.3)	40.79 ± 29.55 (0.23–245.8)	0.0749
CD16+ CD56+ NK cells (% of mononuclear cells)	0.152 ± 0.093 (0.000–1.760)	0.012 ± 0.005 (0.000–0.038)	0.5042
CD16+ CD56+ NK cells (cells/mg)	0.132 ± 0.058 (0.000–0.910)	0.071 ± 0.040 (0.000–0.280)	0.9077

Collected sample volumes and frequencies of the different cell populations are shown as the percentage of mononuclear cells and cell concentration (total cell counts per μL or μg) for SM. Values are shown as mean ± SD (range). The Mann–Whitney test was used for analysis of mononuclear cell frequencies between study groups. All reported p-values are two-tailed. A *p*-value <0.05 was considered to show a statistically significant difference. Significant differences are indicated with asterisks: * *p* < 0.05.

## Supplementary Materials

The following are available online at https://www.mdpi.com/2077-0383/8/10/1631/s1, Figure S1: Cytokine expression comparison between Hip and Knee OA in patients with K&L score 3 osteoarthritis.
