# Supplementary material for: Divergent Mononuclear Cell Participation and Cytokine Release Profiles Define Hip and Knee Osteoarthritis
Source: J Clin Med. 2019 Oct 5;8(10):1631. doi: 10.3390/jcm8101631 (PMC6832735; doi:10.3390/jcm8101631)
Supplement: Supplementary file 1 [file jcm-08-01631-s001.pdf]

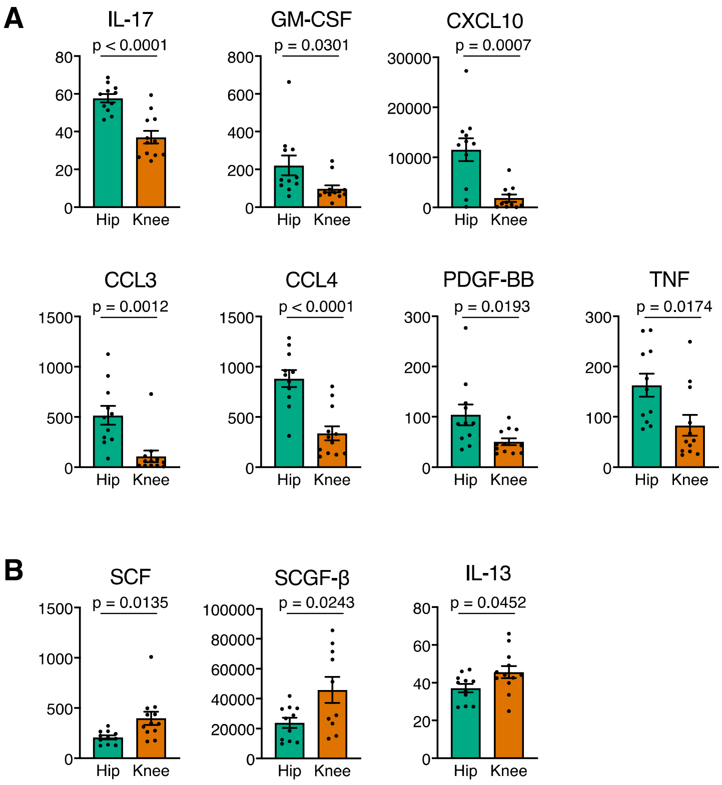

Figure S1. Cytokine expression comparison between Hip and Knee OA in patients with K&L score 3 osteoarthritis.
